# GWIPS‐viz as a tool for exploring ribosome profiling evidence supporting the synthesis of alternative proteoforms

**DOI:** 10.1002/pmic.201400603

**Published:** 2015-04-23

**Authors:** Audrey M. Michel, Anna M. Ahern, Claire A. Donohue, Pavel V. Baranov

**Affiliations:** ^1^ School of Biochemistry and Cell BiologyUniversity College CorkCorkIreland

**Keywords:** Bioinformatics, Non‐AUG initiation, ribo‐seq, Ribosome profiling, Translatomics, uORF

## Abstract

The boundaries of protein coding sequences are more difficult to define at the 5′ end than at the 3′ end due to potential multiple translation initiation sites (TISs). Even in the presence of phylogenetic data, the use of sequence information only may not be sufficient for the accurate identification of TISs. Traditional proteomics approaches may also fail because the N‐termini of newly synthesized proteins are often processed. Thus ribosome profiling (ribo‐seq), producing a snapshot of the ribosome distribution across the entire transcriptome, is an attractive experimental technique for the purpose of TIS location exploration. The GWIPS‐viz (Genome Wide Information on Protein Synthesis visualized) browser (http://gwips.ucc.ie) provides free access to the genomic alignments of ribo‐seq data and corresponding mRNA‐seq data along with relevant annotation tracks. In this brief, we illustrate how GWIPS‐viz can be used to explore the ribosome occupancy at the 5′ ends of protein coding genes to assess the activity of AUG and non‐AUG TISs responsible for the synthesis of proteoforms with alternative or heterogeneous N‐termini. The presence of ribo‐seq tracks for various organisms allows for cross‐species comparison of orthologous genes and the availability of datasets from multiple laboratories permits the assessment of the technical reproducibility of the ribosome densities.

**Abbreviations**
CDS: coding sequence
ribo‐seq: ribosome profiling
TIS: translation initiation site
uORF: upstream ORF
3′ UTR: 3′ untranslated terminal region

Usually only a single translation initiation site (TIS) is annotated for each protein coding ORF in the mRNAs of most eukaryotic organisms. However, due to leaky scanning 1, downstream AUG codons are also frequently used as TISs, see 2 for a review. Proteins produced as a result of such translation are truncated at the N‐terminal ends in comparison with annotated protein sequences. Alternative TISs may also be found upstream of annotated TISs, for example, when translation initiation takes place on upstream in‐frame AUG codons. This situation, however, is very rare since the first in‐frame AUG codon is usually annotated as the TIS. More frequently, proteoforms with N‐terminal extensions are not detected because non‐AUG codons are used for initiation such as CUG 3, or when non‐AUG initiation is modulated by RNA structures formed by nucleotide repeats 4, 5, 6, or when they involve tRNAs other than initiator tRNA‐Met 7, or if initiation takes place in the ribosome A site as in some viral mRNAs 8, 9. The functionality of many N‐terminal extensions produced by such initiation events is often strongly supported by the signatures of protein evolution provided with phylogenetic analysis 10. A remarkable example occurs in the tumor suppressor *PTEN*, where an N‐terminal extension allows an alternative *PTEN* proteoform to be secreted 11. The diversity and complexity of translation initiation mechanisms complicates the detection of TISs when based purely on sequence analysis even in the presence of phylogenetic data.

Ribosome profiling (ribo‐seq) 12 is a promising experimental technique that helps to identify TISs that are operative under given conditions. The technique is based on using translation elongation inhibitors, RNA digestion, and massively parallel sequencing of ribosome protected mRNA fragments followed by their alignment to reference sequences (see 13, 14 for reviews). Attempts to enrich ribosomes at TIS locations have been done using specific drugs that block ribosomes before the formation of the first peptide bond 15, 16 or by a combination of drugs 17, 18. While useful, these techniques suffer from high levels of signal noise, for example, elongating ribosomes pausing at particular codons 19, 20. Therefore, the use of ribo‐seq data is particularly powerful in combination with other techniques, for example, phylogenetic approaches 10 or MS 21, 22, 23.

The GWIPS‐viz (Genome Wide Information on Protein Synthesis visualized) browser 24 is a specialized ribo‐seq browser available at http://gwips.ucc.ie. At present it provides access to the genomic alignments of public ribo‐seq reads in conjunction with mRNA‐seq reads along with relevant annotation tracks. Thus, GWIPS‐viz is a powerful tool for researchers seeking supporting ribo‐seq evidence for alternative proteoforms inferred from phylogenetic analysis or detected with proteomics or other experimental techniques. The GWIPS‐viz genome browser is based on the University of California Santa Cruz (UCSC) Genome Browser (http://genome.ucsc.edu/) 25, but it is specifically tailored for the visualization of ribo‐seq data. The alignments are visualized in two modes. One mode is a coverage plot, where the number of sequence reads aligning to each coordinate is displayed as a bar. Coverage plots are provided for both ribo‐seq data and mRNA‐seq data. The other mode (profile) is used to display the inferred positions of the decoding ribosomes. For the eukaryotic datasets, the coordinates of the A‐site (for elongating ribosomes) or the P‐site (for initiating ribosomes) are inferred for each sequence read by adding a specific offset to the coordinate of the most 5′ nucleotide of the read. For the prokaryotic datasets, a center‐weighted approach 26 is used to indicate the most probable positions of the ribosome A‐site. It needs to be noted that this approach predicts the most likely location of the A‐site, rather than its real location, because the exact length of the footprints as well as the symmetry of the regions flanking the A‐site location are influenced by sequence dependent interactions between ribosomal RNA and mRNA 27. In the current color scheme in GWIPS‐viz, elongating ribosomes data are shown as red, initiating ribosomes as blue, and mRNA‐seq data as green.

Separate tracks for each ribo‐seq study enable cross‐study and even cross‐species comparisons. In addition, to improve the overall signal, we have aggregated ribo‐seq data from several studies into *Global aggregate* tracks. These cumulative global tracks are currently provided for human, mouse, yeast, zebrafish and nematode and new datasets are integrated when available. We would recommend using the global tracks for initial exploration of translated regions, while the individual study tracks can be consulted for more detailed examination.

In the following section, we explain in detail how to use GWIPS‐viz to manually examine available ribo‐seq data for supporting evidence for the synthesis of proteoforms extended at the N‐terminus and how to prepare publication quality screenshots. For this purpose, we use the human *STARD10* gene for which phylogenetic data have been predicted to encode a GUG‐initiated proteoform with an N‐terminal extension 10:

1. Under the *Genomes* page of GWIPS‐viz, select your organism of interest in the genome drop‐down menu (human in this case) and in the gene search box enter the gene symbol (*STARD10* in this example).
2. Scroll down to the following track sections *ribosome profiles*—*initiating ribosomes, ribosome profiles*—*elongating ribosomes* and *mRNA‐seq coverage plots* and set the corresponding *global aggregate* track to full. In addition, set the *base position* and *Refseq genes* tracks from the *annotation tracks* group to full.
3. There is one RefSeq 28 transcript variant for the human *STARD10* gene (see Fig. 1A). The transcript representation is inherited from the UCSC Genome Browser, that is, exons are shown as blocks connected by introns represented as lines with arrowheads displaying the direction of transcription/translation. The annotated coding sequences (CDSs) are shown as thicker blocks than the rest of the transcript exons. The annotated start codon for STARD10 is located in the second exon.
  **Figure 1:**
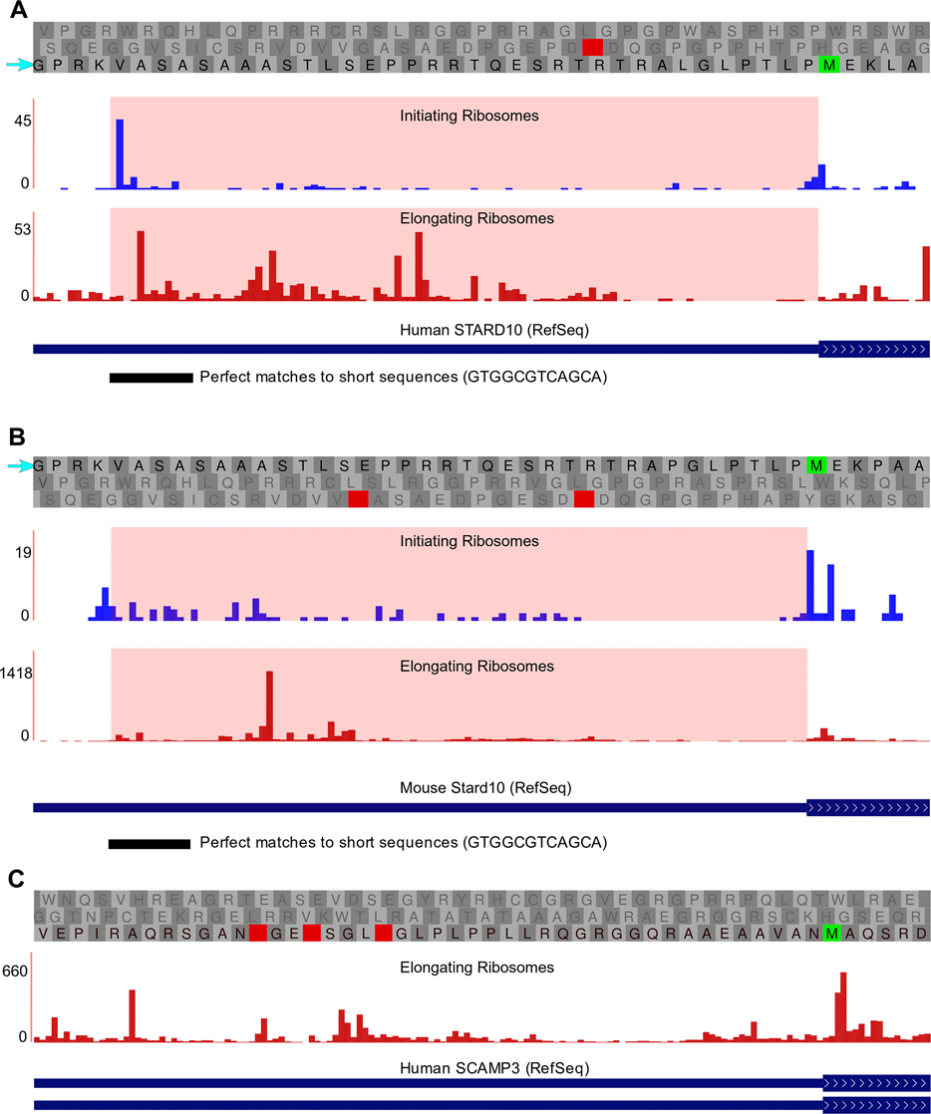
(A) Initiating (blue) and elongating (red) ribosomes profiles for the second exon of the human *STARD10* gene. The region of the predicted GUG‐initiated CDS extension 10 is highlighted in pale pink. In the reading frame organization, AUG codons are represented by green boxes and stop codons are represented by red boxes. The blue arrow points to the reading frame of the CDS extension. Also displayed is the *short match* detected location of the GUG start codon and part of its 3′ flanking nucleotide sequence. The initiating ribosome profile was generated using the *Global aggregate* track in GWIPS‐viz (http://gwips.ucc.ie) using data from four studies 15, 17, 18, 29. The elongating ribosomes profile was generated using data aggregated from 13 studies 15, 17, 18, 29, 38. (B) Initiating ribosomes profile and elongating ribosomes profile for the second exon of the mouse *Stard10* gene. The description is the same as for panel A except that the initiating ribosomes profile was generated from two studies 15, 18 and the elongating ribosomes profile from five studies 15, 16, 37, 39, 40. (C) Elongating ribosomes profile for the first exon of the human *SCAMP3* gene. The ribosome footprints in the region upstream of the annotated AUG start codon (highlighted in pale pink) cannot originate from the translation of an extended CDS as there are three upstream stop codons in the same reading frame. The profile was generated from the same studies as panel A.
4. To zoom into the region of interest (the second exon in this case), click and hold the left mouse button on one edge of the second exon in the *base position* track and drag the mouse to the other edge of the second exon and then release the mouse button.
5. To display the location of the predicted TIS, click on the *short match* track link in the *annotation tracks* group and in the *short (2–30 base) sequence* search box on the *perfect match to short sequence* page, enter “GTG” followed by a string of nucleotides, which is long enough to be unique within the displayed locus (in our case GTGGCGTCAGCA—the location of the predicted TIS is annotated in the supplementary material of 10). Note that since GWIPS‐viz uses DNA sequence as a reference, sequences of DNA rather than RNA nucleotides should be used, for example, GTG for GUG. Also note that the *short match* feature only works for nucleotide sequences. After entering the sequence, set the display mode to full and click submit. The location of this sequence should be displayed as a black block in the corresponding track (see Fig. 1A). An alternative approach is to configure the *base position* track from the *annotation tracks* group to display the sequence of interest. Click on the *base position* link and enter the sequence in the *Motifs to highlight* search box in the configuration window of the track. Note that in order to see the sequence highlighted, the zoom should be at the base level.
6. To explore ribo‐seq data from the individual studies that contribute to the *Global Track*, additional tracks can be displayed.

As can be seen in Fig. 1A, which displays the second exon of the human *STARD10* locus, there is an enrichment of initiating ribosomes at the location of the predicted GUG TIS (blue profile), while elongating ribosomes (red profile) span the entire downstream region up to, and past, the annotated AUG start codon (highlighted in pale pink in Fig. 1A).

The availability of data for several organisms allows us to explore whether the corresponding region is translated in orthologs of the human *STARD10* gene. The display in Fig. 1B is generated by a similar procedure to above, but for the mouse *Stard10* gene. Thus, Fig. 1A and B suggests that the synthesis of the N‐terminal extended products of *STARD10* and *Stard10* is a conserved feature.

How to determine if the footprints aligning to a 5′ leader region originate from ribosomes translating the same ORF as the one to which the annotated CDS belongs or from ribosomes translating a different ORF? The answer to this question may be determined with subsequent analysis of the triplet periodicity within the corresponding region 41, 42. However, even in the absence of periodicity analysis, a simple examination of the ORF organization could rule out the possibility of an extended CDS. In both the human *STARD10* gene and the mouse *Stard10* gene, there is an absence of stop codons in the reading frame upstream of the annotated start codon (Fig. 1A and B). Hence, it is possible that footprints are generated from the same ORF as that of the annotated CDS. Contrast this with the sequence of the first exon of the human *SCAMP3* gene for which ribo‐seq data are displayed in Fig. 1C. The stop codons in the same reading frame upstream of the annotated start codon rule out the possibility of an extended CDS and so the footprints do not belong to the same ORF as the annotated CDS. Instead the ribosome footprints likely originate from an overlapping upstream ORF (uORF) as reported earlier, see Supporting Information of 41.

GWIPS‐viz can also be used for the identification of TISs responsible for the synthesis of proteoforms truncated at the N‐terminus, particularly when initiation at the downstream TIS is substantially more efficient. This is the case with the human *PRKAA1* gene for which the synthesis of a truncated proteoform has been validated previously at the protein level 15, 22. Figure 2A and B shows ribo‐seq data and mRNA‐seq data aligned to the first exon of the human *PRKAA1* gene and the mouse *Prkaa1* gene, respectively. In both cases, the density of elongating ribosomes (red) in the region between the first and the second AUG (highlighted in pale blue) is much lower than downstream of the second AUG. This suggests that the second AUG corresponds to a more efficient TIS. In addition, there is an enrichment of initiating ribosomes at the second AUG (blue plot). Interestingly, the mRNA‐seq density is distributed in a similar manner and therefore it is likely that the reason is not leakiness of the first AUG, but due either to a misannotation of the transcription start site and the translation start site in the current databases or due to an alternative transcript isoform truncated at the 5′ end in comparison with the RefSeq transcript either as a result of alternative transcription initiation or post‐transcriptional processing of the 5′‐end.

**Figure 2:**
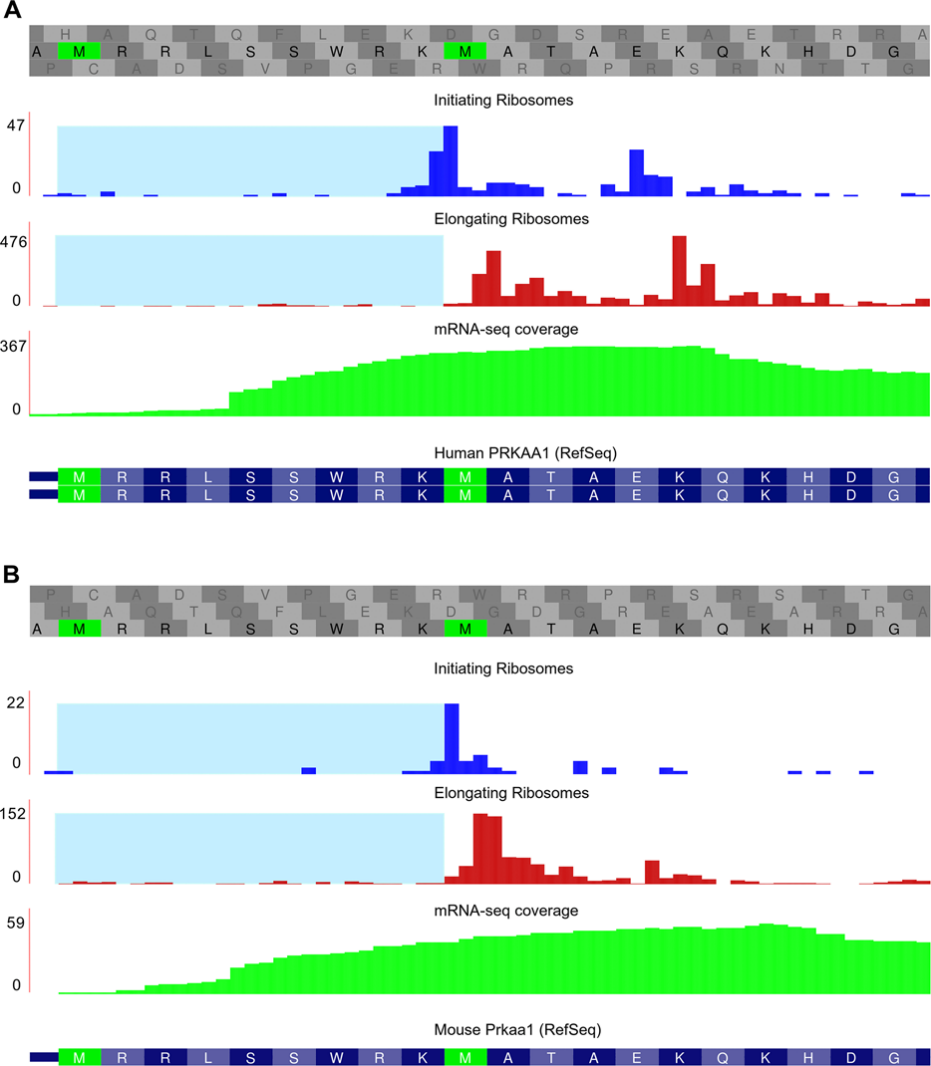
(A) Ribosome profiles and mRNA‐seq coverage plot for the first exon of the human *PRKAA1* gene. The density of elongating ribosomes (red profile) in the predicted CDS truncated region is very low (highlighted in pale blue), while there is an increase in density downstream of the second AUG codon. In addition, there is an enrichment of initiating ribosomes (blue profile) at the second AUG. The mRNA‐seq coverage plot (green) suggests an alternative transcript isoform truncated at the 5′ end. The ribosomes profiles were generated from the same studies as in Fig. 1A and the mRNA‐seq coverage plot was generated using data from seven studies 29, 32, 33, 34, 35, 36, 37 (B) Ribosome profiles and mRNA‐seq coverage plot for the first exon of the mouse *Prkaa1* gene. The description is the same as for panel A except that the ribosome profiles were generated from the same studies as in Fig. 1B and the mRNA‐seq coverage plot was generated using data from four studies 16, 37, 39, 40.

In addition to facilitating the exploration and authentication of truncated or extended proteoforms, GWIPS‐viz can be used to identify or corroborate the translation of ORFs alternative to the one corresponding to the CDS. Figure 3A and B shows ribo‐seq data aligned to the locus of *SLC35A4* in human and *Slc35a4* in mouse, respectively. Most of the ribo‐seq density occurs in a uORF of the corresponding transcripts rather than its annotated CDS. The product of this uORF (MADDKDSLPKLKDLAFLKNQLESLQRRVEDEVNSGVGQDGSLLSSPFLKGFLAGYVVAKLRASAVLGFAVGTCTGIYAAQAYAVPNVEKTLRDYLQLLRKGPD for *SLC35A4* in human and MADDKDSLPKLKDLTFLKNQLERLQQRVEGEVNSGVGQDGSLLSSPFFKGFLAGYVVAKLRASAVLGFAVGTCTGIYAAQAYAVPNVEKALKNYFRSLRKGPD for *Slc35a4* in mouse) was detected earlier with MS 43, 44. Phylogenetic evidence also strongly supports the functional importance of this uORF product in addition to the uORF's regulatory role in providing stress resistance to the translation of the annotated CDS 45. The detection of a product translated from an ORF in the 3′UTR of the human *CHTF8* gene has also been reported 43, 44. Indeed in GWIPS‐viz, a high ribosome occupancy can be seen in the 3′UTR region of the human *CHTF8* transcripts (Fig. 3C) as well as for the mouse orthologs (Fig. 3D). These ribosomes likely originated from the translation of this downstream ORF that is located in an alternative reading frame to the canonical ORF of this gene. This, however, does not necessarily indicate that *CHTF8* mRNA is bicistronic as it is possible that the annotated CDS and downstream ORF are translated in different transcripts originated at the *CHTF8* locus. Indeed a number of alternative transcripts exist among Ensembl 46 genes (including transcript ENST00000306585 shown for human in Fig. 3C and transcript ENSMUST00000169312 shown for mouse in Fig. 3D) in addition to the Refseq 28 transcripts.

**Figure 3:**
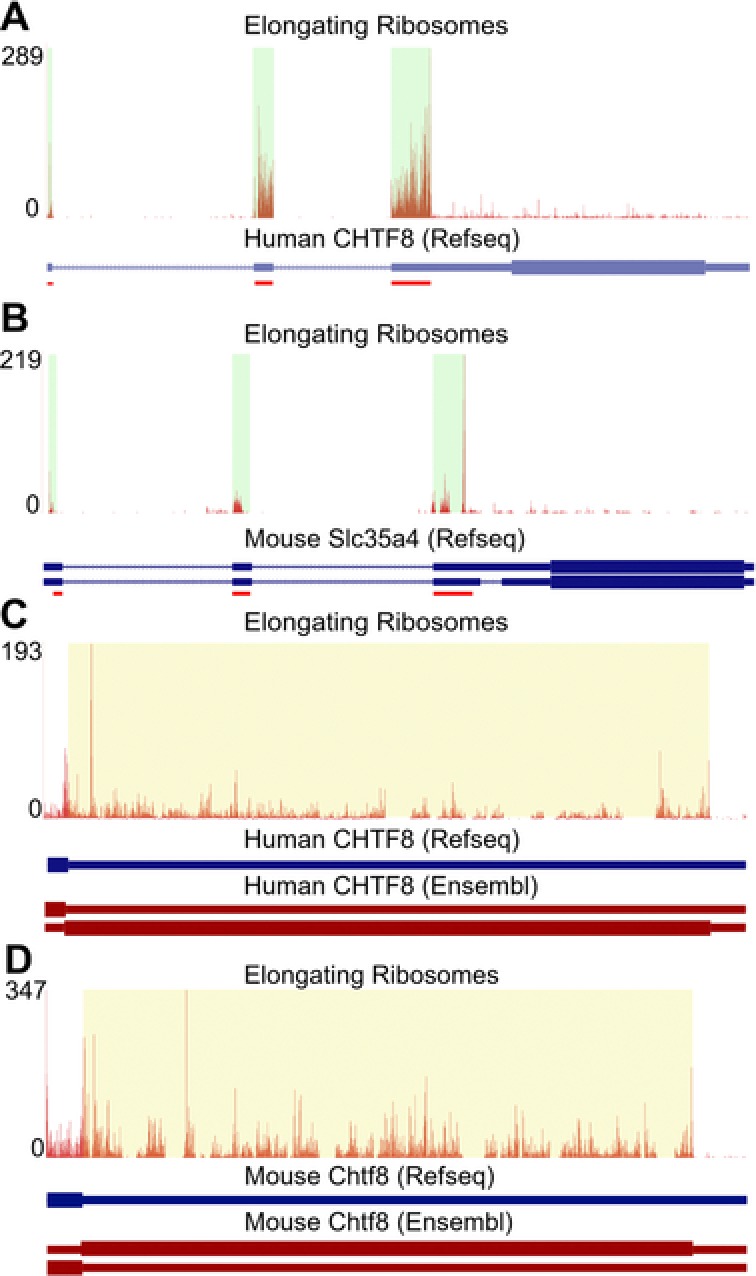
(A) Elongating ribosomes profile for the human *SLC35A4* gene. The density of ribosome footprints in the 5′ leader region (highlighted in pale green) is clearly much higher than in the predicted CDS (the lighter blue color of the RefSeq mRNA transcript indicates that this transcript annotation is “provisional”). The red lines under the RefSeq transcript indicate the location of the predicted uORF. The profile was generated from the same studies as in Fig. 1A. (B) Elongating ribosomes profile for the mouse *Slc35a4* gene. The profile was generated from the same studies as in Fig. 1B. (C) Elongating ribosomes profile for the last exon of the human *CHTF8* gene. There is clearly a high ribosome occupancy (highlighted in pale yellow) in the 3′UTR region of this gene. An alternative Ensembl transcript for this locus contains a CDS that corresponds exactly to the location of the ribosome footprints in the highlighted region. The profile was generated based on the data from the same studies as in Fig. 1A. (D) Elongating ribosomes profile for the last exon in the mouse *Chtf8* gene. The description is the same as for panel C except that the profile was generated from the same studies as in Fig. 1B.

Newly published ribo‐seq datasets are continuously added to GWIPS‐viz. Researchers can also explore their own ribo‐seq data by using the *add custom tracks* functionality. The custom track is only visible to the owner and it allows the researcher to explore their own data in the context of other published datasets. Snapshot figures of the ribosome profiles can be generated using the *PS/PDF* link in the top menu bar and the alignment count data for all tracks are available to download from the *Tables* link for defined regions or for the entire genome.

It is clear that the ribo‐seq technique has a number of limitations in relation to the prediction of alternative proteoforms. The technique does not allow for the discrimination between reads produced from alternative transcript isoforms if they share the same sequences. Often it can be difficult to detect precisely which ORF is being translated due to the convolution of the triplet periodicity signal that occurs when several short overlapping ORFs are translated simultaneously. Nonetheless, as we demonstrated, GWIPS‐viz can be used as a powerful support tool for predictions based on other approaches as well as for generating hypotheses that can be tested using methods other than ribo‐seq.

*The authors have declared no conflict of interest*.
